# Antimicrobial Impact of Different Air-Polishing Powders in a Subgingival Biofilm Model

**DOI:** 10.3390/antibiotics10121464

**Published:** 2021-11-28

**Authors:** Johannes-Simon Wenzler, Felix Krause, Sarah Böcher, Wolfgang Falk, Axel Birkenmaier, Georg Conrads, Andreas Braun

**Affiliations:** 1 Department of Operative Dentistry, Periodontology and Preventive Dentistry, Rheinisch-Westfälische Technische Hochschule University Hospital, Pauwelsstrasse 30, 52074 Aachen, Germany; fkrause@ukaachen.de (F.K.); sboecher@ukaachen.de (S.B.); anbraun@ukaachen.de (A.B.); 2 Center for Dental Microbiology, Bergstr. 26, 24103 Kiel, Germany; wolfgang.falk@web.de (W.F.); a.birkenmaier@odm-kiel.de (A.B.); 3 Division of Oral Microbiology and Immunology, Department of Operative Dentistry, Periodontology and Preventive Dentistry, Rheinisch-Westfälische Technische Hochschule University Hospital, Pauwelsstrasse 30, 52074 Aachen, Germany; gconrads@ukaachen.de

**Keywords:** air polishing, subgingival, biofilm, trehalose, glycine

## Abstract

Subgingival air-polishing devices (SAPD) can reduce bacterial biofilms and thus support periodontal healing. The authors of this study evaluated the effectiveness of the glycine-based and trehalose-based air-polishing powders in removing pathogenic bacteria in a subgingival biofilm model. We treated 56 subgingival pockets in porcine jaws with SAPD. Subgingival air polishing was performed in three groups of 13 pockets each: I, glycine-based powder; II, trehalose-based powder; and III, water alone. Another group (IV) served as untreated controls. Prior to air polishing, inoculated titanium bars were inserted into the pockets containing periopathogenic bacteria such as *Porphyromonas gingivalis* and *Tannerella forsythia*. Remaining bacteria were evaluated using real-time PCR. The numbers of remaining bacteria depended on the treatment procedure, with the lowest number of total bacteria in group I (median: 1.96 × 10^6^ CFU; min: 1.46 × 10^5^; max: 9.30 × 10^6^). Both polishing powders in groups I and II (median: 1.36 × 10^7^ CFU; min: 5.22 × 10^5^; max: 7.50 × 10^7^) showed a statistically significantly lower total bacterial load in comparison to both group IV (median: 2.02 × 10^8^ CFU; min: 5.14 × 10^7^; max: 4.51 × 10^8^; *p* < 0.05) and group III (median: 4.58 × 10^7^ CFU; min: 2.00 × 10^6^; max: 3.06 × 10^8^; *p* < 0.05). Both subgingival air-polishing powders investigated can reduce periopathogenic bacteria and thus support antimicrobial therapy approaches in periodontal treatment regimens.

## 1. Introduction

Periodontitis is an inflammatory disease of the tissue surrounding the teeth caused by bacteria in the plaque biofilm resulting in pocket formation in the gum tissue, loss of attachment, bone destruction, and ultimately tooth loss. The main goal in periodontitis therapy is to reduce or eliminate infection, with major clinical objectives such as reducing the probing depth (PD) and increasing the clinical attachment level (CAL) [1].

Mechanical treatment such as subgingival debridement (SD), which attempts to achieve infection-free conditions by removing bacterial deposits in the supragingival and subgingival biofilm, is therefore still the most important part of periodontal treatment [2]. Although the primary goal of subgingival debridement is to remove subgingival biofilm and calculus, the long-term objective is and should always be to maintain stable periodontal conditions by preventing the recurrence or progression of periodontal disease through the constant removal of emerging biofilm [3,4]. Scalers, curettes, various polishing attachments and pastes, ultrasonic (US) scalers, air-polishing devices (APDs), and lasers are therefore available as treatment tools [3].

Today’s APDs can cover a wide range of indications—from tooth cleaning to cavity pretreatment before restorative therapy and preconditioning before orthodontic bracket application or periodontitis therapy [5,6]. The devices are in widespread use, particularly in the field of prophylaxis. Many reports have described their advantages over the use of manual or rotating instruments [7,8], such as often reduced time requirements [8,9,10,11] and the more effective removal of discoloration [12,13,14]. Undesirable side effects that have been reported include emphysema in the soft tissue, abrasive effects on root cement and exposed dentin [15,16,17], gingival irritation [18], and possible recessions on exposed tooth necks [8,19].

Sodium bicarbonate has been commonly used in air-polishing devices since the 1980s [20], but a variety of powder types are available on the dental market today. Depending on the abrasiveness of the powder used, special attention needs to be given to changes in the surface or to hard substance loss, especially on exposed cement or dentin surfaces [21,22]. In contrast to traditional high-abrasion powders, low-abrasion powders based on glycine or trehalose have recently been introduced into the market. These are considered to be suitable for use on denuded root surfaces, as well as in the depth of periodontal pockets, and are thought to provide effective subgingival plaque removal with a minimum abrasion of the root cement and dentin with a putative antimicrobial effect [20,23,24,25]. Air-polishing powder based on glycine, which is a nonessential amino acid, was introduced into the dental market in 2003. In the human body, glycine acts as an inhibitory neurotransmitter and is an essential component of collagen; it is also considered to have anti-inflammatory, immunomodulatory, and cytoprotective effects. Glycine crystals are odorless, colorless, and highly water-soluble; due to its light, sweet taste, glycine is also used as a licensed food supplement (E 640) [26]. With a mean size of less than 45 µm, glycine powder particles are approximately four times smaller than particles of conventional sodium bicarbonate powder [23]. Glycine powder is also suitable for supragingival applications, but its main area of application is for cleaning root surfaces [5,27,28,29], where it allows for effective plaque removal from root cement and dentin without significant damage in vitro [12]. Studies have confirmed that its abrasiveness is 80% lower than that of sodium bicarbonate [30], and less traumatization of the gingival tissue has also been observed in comparison to the use of hand instruments or sodium bicarbonate air polishing [14,31]. In periodontal sites with a probing depth of 3–5 mm, the use of glycine powder has been reported to be even more effective for the removal of subgingival plaque than hand instruments [12]; in comparison with ultrasonic scalers, no difference was detected [11].

A newer, recently introduced powder named trehalose is a highly water-soluble, noncariogenic disaccharide that has been approved for use in food processing [32] and is also suitable for diabetics. With an average particle size of 30–65 µm, it can be used for supragingival and subgingival applications in accordance with the manufacturer’s instructions [25]. The manufacturer’s data from preliminary in vitro trials indicate that the abrasive effect on dental hard substance is comparable to that of glycine, and it has a comparable particle diameter [33]. Only a few studies are to date available on the trehalose air-polishing powder. A study by Kruse et al. reported that subgingival air polishing using trehalose powder showed comparable clinical outcomes (probing depth, clinical attachment level, and bleeding on probing) to subgingival ultrasonic scaling [33]. These results were confirmed by another recently published study by Kruse et al. that also found no statistically significant differences in clinical parameters (probing depth, clinical attachment level, and bleeding on probing) between patients treated with ultrasonic scaling and those receiving air polishing using trehalose powder [25].

The range of applications for modern low-abrasion prophylactic powders has increasingly been extended to include supportive treatment for periodontitis and peri-implant therapy, where APDs can also be used during systematic periodontal therapy and should be regarded as an adjuvant therapy method. Specially designed approaches that allow for the subgingival cleaning of root surfaces up to pocket depths of 10 mm, as well as of furcations or difficult-to-access approximal spaces with low-abrasion powder types [34,35,36], have proven to be beneficial.

To date, only a few published studies are available on trehalose-based air-polishing powders to verify their efficacy for antibacterial purposes during subgingival tooth cleaning. There is consequently no comprehensive evidence available on the use of trehalose as an air-polishing powder. The aim of the present study was therefore to investigate whether there are any differences in effectiveness between glycine-based and trehalose-based polishing powders for bacterial removal in an artificial subgingival biofilm model, testing the hypothesis of the different polishing powders having similar impacts on subgingival bacterial reduction.

## 2. Materials and Methods

### 2.1. Experimental Design

**Figure 1:**
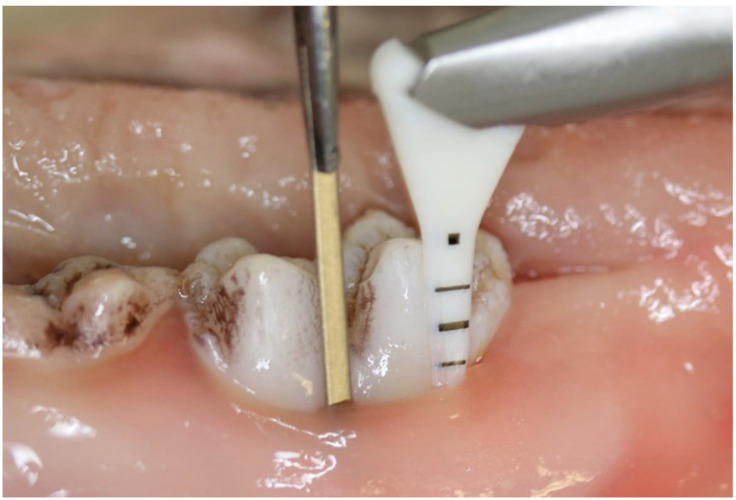
Instrumentation, with both the air-polishing nozzle (**right**; black markings correspond to the nozzle scale) and the biofilm-coated titanium bar (**left**) inserted into the subgingival pocket.

For the purpose of this study, fourteen lower porcine jaws were obtained immediately after slaughter (slaughterhouse waste). Fifty-six subgingival pockets were prepared, and the lower 5 mm of flat titanium bars (1.5 mm wide and 2.5 cm long) were inoculated with an artificial biofilm before being subsequently inserted into the pockets and treated in accordance with their group assignment with a subgingival air-polishing device (Air-Flow Hany Perio, EMS, Nyon, Switzerland) and the corresponding subgingival nozzle (Figure 1).

The polishing device was connected to the dental unit at a constant air pressure of 4000 hPa with a water flow rate of 60 mL/min. One investigator (J.-S.W.) filled the device’s powder chamber to the full level for each use, ensuring that the openings of the tubes were not covered with powder to avoid a clogging effect. The operator (A.B.) was not aware of the study group assignment in each case and performed subgingival air polishing in three groups of 13 pockets each: I, glycine-based powder (Air-Flow Perio, EMS, Nyon, Switzerland); II, trehalose-based powder (Lunos Prophy Powder Perio Combi, Duerr Dental, Bietigheim-Bissingen, Germany); and III, water alone. Group IV consisted of 13 inoculated but untreated titanium bars serving as untreated negative controls.

The biofilm-coated titanium bars (25 × 1.5 × 0.5 mm; made of rolled titanium wire, DENTAURUM, Ispringen, Germany) were created as follows: the starting medium consisted of 500 µL of a thioglycollate suspension from a highly bacterially positive patient sample (according to qualitative PCR results with the selection criterion of as many pathogens as possible) and 500 µL of saliva from voluntary donors. Each of the titanium bars (purified using isopropanol and autoclaving) had 4 µL of this suspension added to it and was fixed in a closed box overnight at 37 °C. This procedure was carried out four times (resulting in a total of 16 µL of suspension per test bar). The limit of bacterial coating here was naturally limited by the width of the titanium bars. The described coating contained common periopathogenic bacteria such as *Porphyromonas gingivalis* (Pg), *Tannerella forsythia* (Tf), *Treponema denticola* (Td), *Fusobacterium nucleatum* (Fn), *Parvimonas micra* (Pm), *Campylobacter rectus* (Cr), *Eikenella corrodens* (Ec), *Aggregatibacter actinomycetemcomitans* (Aa), and *Prevotella intermedia* (Pi).

In all groups, the nozzle (Perio-Flow Nozzle, EMS, Nyon, Switzerland) of the handpiece was first inserted to the bottom of the pocket, where the air-polishing device was then activated in a constant sweeping motion. The application time of 10 s per sample was measured using a stopwatch; water settings were exactly identical in all of the groups. Rinsing with water alone (group III) in the same experimental conditions served as a mechanical control. Cross-contamination between the individual samples and groups was prevented by using a fresh nozzle for each sample.

After air polishing, the samples were transferred into Eppendorf tubes, and the bacterial load was quantified by a specialized external laboratory (Oro-Dental Microbiology ODM, Kiel, Germany) using a real-time quantitative polymerase chain reaction (RT qPCR). A TaqMan Assay, that had been validated with the micro-IDent plus test system, was then applied. The main parameter for analysis was the total bacterial load (TBL; either of all bacteria or individual pathobionts) given as colony-forming units (CFU). This was determined by the basic assumption that the 16S rRNA target gene was present only once in each species, despite the fact that different bacterial genera have different 16S operon numbers. A ten-fold dilution series of a molecular standard (corresponding to a certain number of CFUs) was used as the calibration standard for measuring patient samples with unknown contents of bacteria. For measurements, crossing point (Cp) values were determined. Cp values indicate the number of PCR cycles at which the fluorescence exceeds a threshold value significantly higher than the background fluorescence.

### 2.2. Statistical Analysis

A power analysis was performed prior to the study. When the data for the five subgingival pockets actually measured in each group were analyzed, an effect size of 1.32 was estimated. For an alpha error of 0.05 and a power of 0.8, a total sample size of at least eleven specimens in each group was calculated. The normal distribution of the values was assessed using the Shapiro–Wilk test. Since not all data were normally distributed, values for crack formation between the groups were analyzed using a nonparametric test (Kruskal–Wallis) and Mann–Whitney pairwise comparisons. The values were recorded in an Excel data sheet (Microsoft, Redmond, Washington, DC, USA) and afterward analyzed using statistical analysis software (IBM SPSS Statistics for Windows, Version 24.0; IBM Corporation, Armonk, New York, NY, USA). Differences were considered as statistically significant at *p* < 0.05. The sequentially rejective Bonferroni correction of the critical *p* value was used when multiple statistical tests were performed simultaneously on a single dataset. Box plot diagrams are used to show the median, first and third quartiles, and minimum and maximum values (whiskers). Values of more than 1.5–3 times the interquartile range were specified as outliers and marked as data points. Values of more than three times the interquartile range were specified as distant outliers and marked as asterisks.

## 3. Results

**Figure 2:**
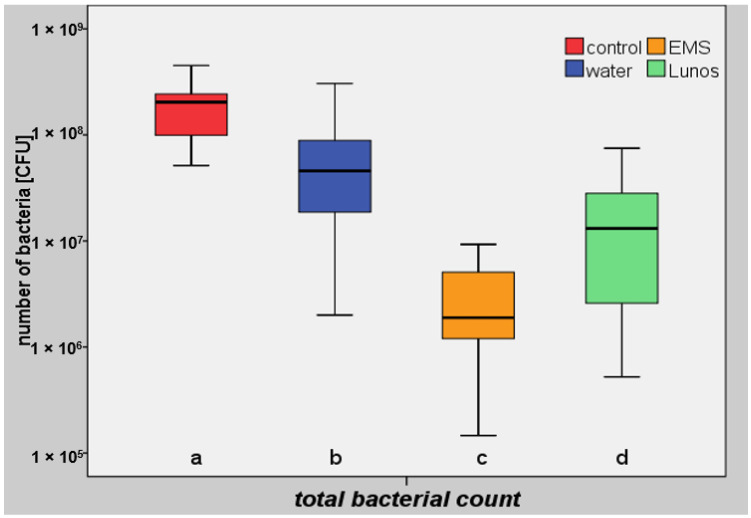
The total bacterial loads in the study groups. Both air polishing powders led to a statistically significantly lower total bacterial load in comparison with the untreated controls and the “water alone” group (*p* < 0.05)) (Table 2). The letters a–d show statistically significant differences. CFU, colony-forming units; EMS, glycine (Air-Flow Perio); Lunos, trehalose (Lunos Prophy Powder Perio Combi).

The number of bacteria remaining after air polishing was dependent on the treatment procedure used, with the lowest number of total bacteria being seen in group I (median: 1.96 × 10^6^ colony-forming units (CFUs); min: 1.46 × 10^5^; max: 9.30 × 10^6^; interquartile range (IR): 3.22 × 10^6^) (Figure 2). The two polishing powders in groups I and II (median: 1.36 × 10^7^ CFUs; min: 5.22 × 10^5^; max: 7.50 × 10^7^; IR: 2.34 × 10^7^) showed a statistically significantly lower total bacterial load in comparison to both the untreated controls (median: 2.02 × 10^8^ CFUs; min: 5.14 × 10^7^; max: 4.51 × 10^8^; IR: 1.37 × 10^8^; *p* < 0.05) and in comparison to group III with water alone (median: 4.58 × 10^7^ CFUs; min: 2.00 × 10^6^; max: 3.06 × 10^8^; IR: 6.17 × 10^7^; *p* < 0.05) (Table 1 and Table 2).

**Figure 3:**
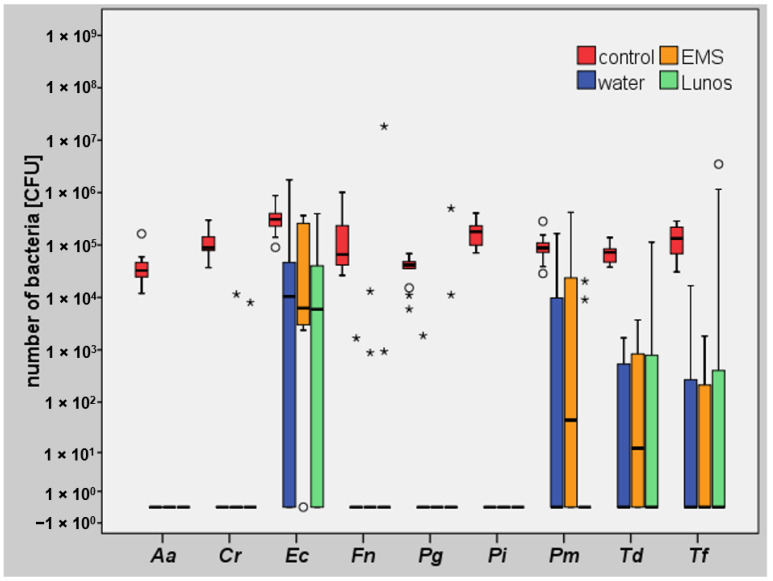
Colony-forming units (CFUs) of periopathogenic bacteria in the study groups. With similar amounts of bacteria at the beginning, the strongest reductions were observed for *P. gingivalis* (Pg), *F. nucleatum* (Fn), *C. rectus* (Cr), *A. actinomycetemcomitans* (Aa), and *P. intermedia* (Pi). EMS, glycine (Air-Flow Perio); Lunos, trehalose (Lunos Prophy Powder Perio Combi); mild outliers: ° and extreme outliers: *.

In relation to the median initial bacterial quantity, a bacterial reduction of 77.6% was observed when only water was used without an air-polishing powder. When the trehalose-containing powder was used, the percentage reduction relative to the median was 93.3%, and the reduction in the glycine group was 99.0%. Though the initial quantities of periodontal pathogenic bacteria were similar at baseline, a reduction to a median bacterial load of 0 CFU (under the detection limit, respectively) after air polishingwas only observed for *Porphyromonas gingivalis* (Pg), *Fusobacterium nucleatum* (Fn), *Campylobacter rectus* (Cr), *Aggregatibacter actinomycetemcomitans* (Aa), and *Prevotella intermedia* (Pi) in all of the studied groups (Figure 3).

## 4. Discussion

The results of the present study confirm the study hypothesis that the use of different polishing powders have similar effects on subgingival bacterial reduction. Since the introduction of air-polishing devices and powders into routine practice, several studies have closely investigated the advantages and disadvantages of treatment with them. Although mechanical treatment in the form of subgingival debridement is still the most important part of periodontal disease treatment, its frequent use is often associated with adverse effects that can involve damage to the periodontium, enhanced hard substance loss, gingival recessions, and hypersensitive tooth necks [9,11,16,34,35,36].

Because new biofilm forms rapidly on treated root surfaces after subgingival instrumentation [6,37], following the pretreatment composition of the subgingival microflora [6,38], repeated subgingival debridement as part of supportive periodontal therapy appears to be necessary in order to maintain long-term stable conditions [6,39]. Since the formation of subgingival calculus is a very slow process in contrast to supragingival calculus and little to no subgingival calculus appears to develop within 3–6 months after treatment [6,40], its presence should not normally be expected in patients who are participating in supportive periodontal therapy programs [40,41,42].

As a result of the differing characteristics of nonmineralized bacterial episodes, adverse effects of mechanical treatment may increasingly take place. It has therefore been suggested that using air-polishing devices (APDs) as a gentle form of therapy to complement traditional subgingival debridement may reduce the accumulated adverse effects of frequent instrumentation [26]. Specific benefits are seen in relation to the efficacy of APDs, their low abrasiveness and the resulting reduction in the loss of hard tissue in comparison with curettage or the use of ultrasound-assisted systems [7,8].

However, air-polishing devices that use powders approved for subgingival application are not as effective with heavy deposits and are not able to remove subgingival calculus on their own, so the exclusive use of them in periodontal therapy may be limited [16]. This is why most studies have strongly emphasized the view of APDs as an adjuvant treatment method following the removal of mineralized bacterial deposits using conventional subgingival debridement [26]. This is the main reason why there has been a search for new low-abrasion powders for subgingival application that are equally effective [33].

The safety of conventional sodium bicarbonate air-polishing powder has been established for applications on intact enamel surfaces, but the powder has been reported to be unsuitable for use on root surfaces or denuded dentin when a significant loss of hard substance is seen [20]. This has been confirmed by several studies aimed at quantifying root-substance loss caused by air polishing, where defect depths of up to 856 µm have been reported [11,20,43,44,45]. As a result, low-abrasion air-polishing powders based on glycine and trehalose, with mechanical properties that differ from those of standard sodium bicarbonate, have been developed in order to optimize efficacy and enhance the safety of air polishing on dentin or cement [20].

Air-polishing powder based on glycine, which is a nonessential amino acid, was introduced into the dental market in 2003. In the human body, glycine acts as an inhibitory neurotransmitter and is an essential component of collagen; it is also considered to have anti-inflammatory, immunomodulatory, and cytoprotective effects. Glycine crystals are odorless, colorless, and highly water-soluble; due to its light and sweet taste, glycine is also used as a licensed food supplement (E 640) [20,26]. With a mean size of less than 45 µm, glycine powder particles are approximately four times smaller than particles of conventional sodium bicarbonate powder [20]. Glycine powder is also suitable for supragingival applications, but its main area of application is for cleaning root surfaces [6,28,29,46], where it allows for effective plaque removal from root cement and dentin without significant damage in vitro [16]. Studies have confirmed that its abrasiveness is 80% lower than that of sodium bicarbonate [30], and less traumatization of the gingival tissue has also been observed in comparison to the use of hand instruments or sodium bicarbonate air polishing [13,31]. In periodontal sites with a probing depth of 3–5 mm, the use of glycine powder has been reported to be even more effective for the removal of subgingival plaque than hand instruments [16]; in comparison to ultrasonic scalers, no difference was detected [9].

Although they are effective, treatment approaches aiming to reduce subgingival microflora such as mechanical debridement using hand instruments or oscillating scalers are limited by the morphologic conditions on the subgingival root surface—such as concavities, grooves, difficult-to-access furcations, and distal locations [35,47,48] in which bacteria can persist in root cement and dentin tubules—that can also serve as a reservoir for recolonization [49].

The use of APDs may also have additional benefits, in contrast to mechanical debridement instruments, that are effective only on direct contact with the surface being treated and whose effectiveness in narrow grooves and furcations is, in purely technical terms, limited by the size of the required working blade. Since a certain distance to the tooth surface is required for the correct application of APDs, their effectiveness in narrow spaces may not be limited to the same extent. Further studies to evaluate this hypothesis are required and are currently in planning.

Other possible antibacterial effects of different powder types on residual bacteria should also be taken into consideration here and could represent an additional gain. Studies investigating the antibacterial efficacy of air-polishing powders have reported inhibitory activity through the bacteriostatic effects of glycine. Drago et al. reported a reduction of about 30% in surviving cells in residual biofilm after air polishing with glycine [50]. Although some studies have found that microbiological outcomes are not influenced by the method of subgingival debridement, as there were no intergroup differences in the main periodontopathogens or total bacterial load [3,9], Petersilka and co-workers reported that air polishing using glycine powder resulted in a significantly greater reduction in the numbers of subgingival bacteria than hand instrumentation [3,34]. Similarly, Flemmig et al. reported that after the use of glycine powder for subgingival treatment, samples showed significantly fewer colony-forming units in comparison to samples treated using hand instrumentation [6,20]. Investigations of the treatment of titanium surfaces have reported similar findings with regard to bacterial recolonization within the first 24 h after treatment, with significantly better results with the use of glycine than with sodium bicarbonate treatment [51].

Previous findings are in line with the results of the present study because the number of bacteria remaining after air polishing was found to be dependent on the treatment procedure. Both powder types showed a statistically significantly lower total bacterial load in comparison to both the untreated controls (group IV) and samples treated with water flux only (group III). The lowest number of total bacteria, however, was observed in samples treated with glycine (group I), which showed a median percentage reduction of 87.0% in comparison with the untreated controls. A statistically significant difference in bacterial reduction were observed between the two powder types, with trehalose powder (group II) showing a median percentage reduction of remaining bacteria of 75.47%. For greater accuracy, samples treated with water flux alone were used as mechanical controls in order to evaluate the reduction in the bacterial load due to the inherent water jet alone; as a result, the samples already showed a percentage reduction of approximately 17.41% in comparison to the untreated controls.

It should be kept in mind here that in vitro study models can never reflect the complexity of actual in vivo situations. We must mentioned the limitations of the used in vitro study design, including the absence of the aggravating conditions that are present on subgingival tooth surfaces. Since true morphological conditions were not accurately imitated by the used biofilm-covered titanium bars, and the greater complexity of actual root surfaces may impede the removal of biofilm and make antibacterial treatment less effective. The porcine subgingival pocket model used in the present study may provide a much better model of gingival and subgingival pocket formation—as well as access to the depths of periodontal defects in aggravated conditions—than artificial pocket models, but it is not able to accurately simulate difficult and cramped conditions in the oral cavity [1].

Since a large number of bacterial species are involved in the etiology of oral health problems, study designs that use artificial biofilm models may provide a better reflection of clinical conditions than study designs using cultures with single microorganisms. Investigations of multispecies biofilms, as used in the present study, are likely to have greater validity but can still only reflect a part of the true complexity of oral biofilms. The absence of environmental effects may also be mentioned as a limiting factor with in vitro study designs [1].

Slight inaccuracies may have arisen from the movement of the air-polishing nozzle along the specimens performed manually by the operator (A.B.), which, as a human component, could have led to a susceptibility to errors. However, such slight differences were largely avoided by the fact that the entire practical part of the study was carried out by a single person. Nevertheless, such slight inaccuracies in application can be expected in the same way in clinical use on patients [52].

As only a few previous studies on the topic have been published, further investigations into the possible additional advantages of air-polishing powders on residual bacteria in dentin tubules and cement, as well as on possible effects in deeper root dentin layers, are needed.

Patients perceive the use of hand instruments or ultrasonic scalers for subgingival debridement as unpleasant, while the use of air-polishing devices is often reported to be more comfortable and less painful [25,33]. A systematic review of patients’ perception of the use of air-polishing devices during periodontal treatment by Bühler et al. reported that discomfort was consistently equivalent or lower when air-polishing powders consisting of glycine or erythritol were applied in comparison to root surface instrumentation using hand instruments or ultrasonic devices [46]. Since patients’ perceptions, such as experiencing discomfort during a treatment, are an important factor for treatment acceptance—especially during the phase of supportive periodontal therapy in which adequate long-term compliance and active cooperation by patients are required—the use of air-polishing devices may increase or at least maintain patient compliance [46,53].

Finally, it should be noted that air-polishing procedures appear to be less time-consuming than other treatment procedures [11,54]. Using air-polishing devices may therefore be a good alternative to conventional techniques for subgingival biofilm removal [31].

## 5. Conclusions

Both of the subgingival air-polishing powders investigated here can reduce periopathogenic bacteria and thus support antimicrobial therapy approaches in periodontal treatment regimens. 

## Figures and Tables

**Table 1:** Total bacterial count (colony-forming units) in the study groups.

	Group IV (Controls)	Group III (Water)	Group I (Glycine) ^a^	Group II (Trehalose) ^b^
*n*	14	14	14	14
Mean value	2.02 × 10^8^	7.25 × 10^7^	3.12 × 10^6^	2.23 × 10^7^
Standard deviation	1.19 × 10^8^	8.16 × 10^7^	2.97 × 10^6^	2.53 × 10^7^
Median value	2.04 × 10^8^	4.58 × 10^7^	1.96 × 10^6^	1.36 × 10^7^
Maximum value	4.51 × 10^8^	3.06 × 10^8^	9.30 × 10^6^	7.50 × 10^7^
Minimum value	5.14 × 10^7^	2.00 × 10^6^	1.46 × 10^5^	5.22 × 10^5^
Interquartile range	1.37 × 10^8^	6.17 × 10^7^	3.22 × 10^6^	2.34 × 10^7^
Percentage reduction		77.6	99.0	93.3

^a^ Glycine-based powder: Air-Flow Perio (EMS, Nyon, Switzerland). ^b^ Trehalose-based powder: Lunos Prophy Powder Perio Combi (Duerr Dental, Bietigheim-Bissingen, Germany).

**Table 2:** Statistical analysis of the bacterial count (Mann–Whitney test and Bonferroni correction of the critical *p* value).

	Group IV (Controls)	Group III (Water)	Group I (Glycine) ^a^	Group II (Trehalose) ^b^
Controls		0.001019	7.468 × 10^−6^	1.144 × 10^−5^
Water	0.001019		3.917 × 10^−5^	0.02158
Glycine	7.468 × 10^−6^	3.917 × 10^−5^		0.006702
Trehalose	1.144 × 10^−5^	0.02158	0.006702	

^a^ Glycine-based powder: Air-Flow Perio (EMS, Nyon, Switzerland). ^b^ Trehalose-based powder: Lunos Prophy Powder Perio Combi (Duerr Dental, Bietigheim-Bissingen, Germany).
